# miR-2337 induces TGF-β1 production in granulosa cells by acting as an endogenous small activating RNA

**DOI:** 10.1038/s41420-021-00644-4

**Published:** 2021-09-18

**Authors:** Lingfang Wang, Xing Du, Qiqi Li, Wangjun Wu, Zengxiang Pan, Qifa Li

**Affiliations:** College of Animal Science and Technology, Nanjing Agricultural University, 210095 Nanjing, China

**Keywords:** Apoptosis, Growth factor signalling

## Abstract

Transforming growth factor-β1 (TGF-β1) is essential for ovarian function and female fertility in mammals. Herein, we identified three completely linked variants, including two known variants referred to as c.1583A > G and c.1587A > G and the novel variant c.2074A > C in the porcine *TGF-β1* 3′-UTR. An important role of these variants in Yorkshire sow fertility was revealed. Variants c.1583A > G and c.1587A > G were located at the miRNA response element (MRE) of miR-2337 and affected miR-2337 regulation of *TGF-β1* 3′-UTR activity. Interestingly, miR-2337 induces, not reduces the transcription and production of TGF-β1 in granulosa cells (GCs). Mechanistically, miR-2337 enhances *TGF-β1* promoter activity via the MRE motif in the core promoter region and alters histone modifications, including H3K4me2, H3K4me3, H3K9me2, and H3K9ac. In addition, miR-2337 controls TGF-β1-mediated activity of the TGF-β signaling pathway and GC apoptosis. Taken together, our findings identify miR-2337 as an endogenous small activating RNA (saRNA) of *TGF-β1* in GCs, while miR-2337 is identified as a small activator of the TGF-β signaling pathway which is expected to be a new target for rescuing GC apoptosis and treating low fertility.

## Introduction

Transforming growth factor-β1 (TGF-β1) is an essential ligand of the TGF-β signaling pathway [1, 2]. As an extracellular signal molecule, the TGF-β1 signal first binds to the TGF-β receptors on the cell membrane, thereby activating R-SMADs in the cytoplasm, and ultimately enters the nucleus with Co-SMAD to control multiple cell functions [3]. TGF-β1 plays a vital role in cell growth, proliferation, and apoptosis of various normal cell types, as well as pathological cell types [1, 4]. In the ovary, TGF-β1 is highly expressed, as it is secreted from various follicular cells, and is detected in follicular fluid [5–7]. Increasing evidence has demonstrated that TGF-β1 is closely involved in ovarian functions, including follicular development [8, 9], ovulation [10], steroid production [11, 12], and GC apoptosis [13]. For instance, in female mice with knock out of the *TGF-β1* gene, ovarian function was destroyed and the oocytes decreased by 40% [7]. TGF-β1 promotes estradiol release from mouse GCs [14], and inhibits the production of progesterone and prostaglandin E2 in the corpus luteum from bovine ovaries [15]. Our recent report demonstrated that TGF-β1 can inhibit porcine GC apoptosis [13]. In addition, TGF-β1 is involved in female fertility, including fertilization, early embryo development and litter size [2, 7].

As a multifunctional cytokine that is essential for mammalian health and disease, TGF-β1 regulation has always been a key focus of attention. Evidence suggests that *TGF-β1* is regulated by various genetic and epigenetic factors [16, 17]. For genetic factors, more than a dozen mutations, including single nucleotide polymorphisms such as rs1800470 (C29T) and rs1800471 (G74C), and deletion/insertion polymorphisms such as c.−2389_−2391insAGG, as well as several transcription factors such as AP1 and SP1, have been demonstrated to control TGF-β1 levels in various tissues and cell types [18, 19]. The T allele at variant C29T is a risk factor for genetic susceptibility to myocardial infarction, and individuals with genotype TT showed low TGF-β1 concentration in the serum [20]. For epigenetic factors, DNA methylation, histone modification factors, and non-coding RNAs (ncRNAs) all control TGF-β1 expression [17, 21, 22]. Myeloid-derived suppressor cells with high *TGF-β1* mRNA levels are characterized by low methylation of CpG islands (CGIs) in the promoter region, whereas antigen-presenting cells with low *TGF-β1* mRNA levels exhibit high methylation of CGI [17]. Recently, microRNAs (miRNAs) have emerged as new regulators of *TGF-β1*, such as the H19 and miR-29b-3p axis [23], and the RNF7 and miR-543 axis [22]. However, as an important regulator of follicular development in female fertility, miRNA target regulation of the *TGF-β1* gene in follicular cells is largely unknown. Herein, we demonstrate that miR-2337 functions as an endogenous small activating RNA (saRNA) to induce TGF-β1 production and inhibit apoptosis in GCs by directly interacting with miRNA response element (MRE) in the core promoter.

## Results

### Variants in Yorkshire TGF-β1 3′-UTR

**Fig. 1:**
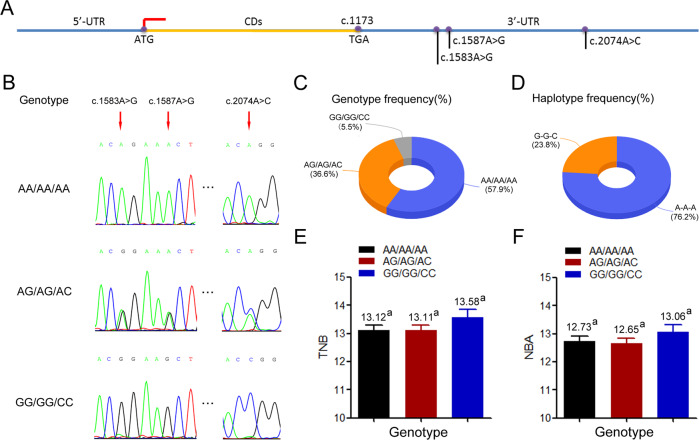
Mutations in *TGF-β1* 3′-UTR and their association analysis with sow reproductive performance. **A** Schematic diagram of the location of SNPs on TGF-β1 mRNA. **B** Peak plot of three genotypes. **C** Frequency of three genotypes. **D** Frequency of haplotypes. **E**, **F** Association analysis between polymorphisms of *TGF-β1* 3′-UTR and TNB trait (**E**), or NBA trait (**F**) in Yorkshire pig population. *n* = 325. Data are represented as the least squares means ± S.E.M.

A previous study demonstrated the involvement of two variants of sow fertility in the Yorkshire *TGF-β1* 3′-UTR [24]. To further investigate the full view of variants in the whole 3′-UTR of porcine *TGF-β1*, we screened this region using pooled-DNA sequencing in multiple pig breeds including Yorkshire, Suhuai, and Erhualian. Three completely linked SNPs, including two known variants termed c.1583A > G and c.1587A > G, and the novel variant c.2074A > C were identified in this region (Figs. 1A and S1). In a Yorkshire population (*n* = 325), there were three genotypes, AA/AA/AA, AG/AG/AC, and GG/GG/CC, of which the AA/AA/AA genotype was the dominant genotype (57.9%) (Fig. 1B, C). Two haplotypes A-A-A and G-G-C were identified in this population, of which A-A-A was the dominant haplotype (76.2%) (Fig. 1D). Association analysis revealed that the TNB of sows with the GG/GG/CC genotype was 13.58, which is 0.47 more than that of the AG/AG/AC genotype and 0.46 more than that of the AA/AA/AA genotype, but the difference was not significant (Fig. 1E). The NBA of sows with the GG/GG/CC genotype was 13.06, which is 0.39 more than that of the AG/AG/AC genotype and 0.33 more than that of the AA/AA/AA genotype, but the difference was not significant (Fig. 1F). This trend is consistent with the results in another Yorkshire population, in which the TNB of sows with the GG/GG genotype for c.1583A > G and c.1587A > G was significantly higher than that of the AA/AA genotype [24]. Taken together, these data indicate an important role that *TGF-β1* 3′-UTR variants play on the reproductive performance of Yorkshire sows.

### Variants in the MRE motif prevent target inhibition of miR-2337 on TGF-β1 3′-UTR

**Fig. 2:**
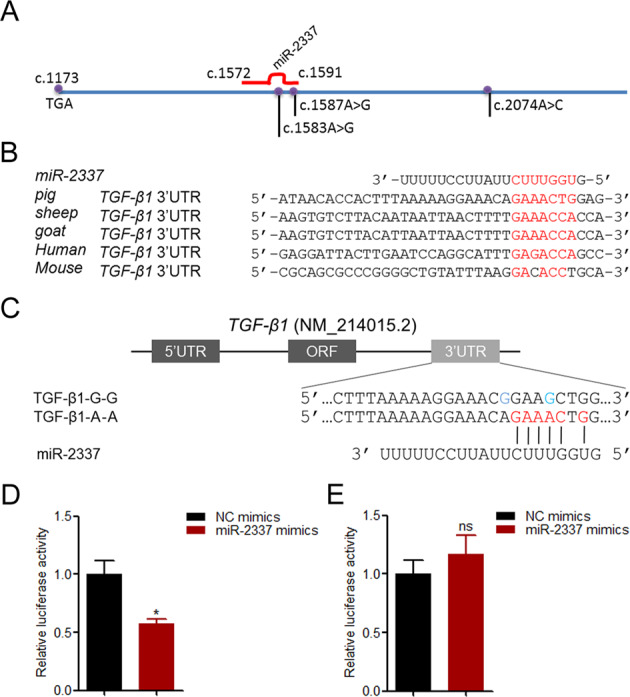
Variants in the MRE motif prevent inhibition of miR-2337 on *TGF-β1* 3′-UTR. **A** Schematic showing the binding site of miR-2337 in the porcine *TGF-β1* 3′-UTR. **B** Alignments of miR-2337 mature sequences and the mammalian *TGF-β1* 3′-UTR. **C** Schematic annotation of the report vector of the *TGF-β1* 3′-UTR containing the MRE motif of miR-2337. MRE motifs carried with haplotypes A-A or G-G were cloned into pmirGLO plasmids. **D**, **E** The effect of miR-2337 on activity of the report vector of the *TGF-β1* 3′-UTR. Report vectors of haplotypes A-A (**D**) or G-G (**E**) were co-treated with miR-2337 mimics, luciferase activity was measured. Data are represented as means ± S.E.M.. (*n* = 3). *p < 0.05.

Variants in MREs usually alter 3′-UTR functioning by influencing the interaction between miRNAs and the 3′-UTR [25]. Notably, two variants c.1583A > G and c.1587A > G were located at the MRE motif of miR-2337 (Figs. 2A, S2, and S3), and mutations led to the loss of the MRE motif. Furthermore, the MRE motif of miR-2337 was also detected in *TGF-β1* 3′-UTR of other mammals (Fig. 2B), indicating that *TGF-β1* is a candidate target of miR-2337 in mammals. To investigate whether miR-2337 targets *TGF-β1*, we generated a reporter construct of the *TGF-β1* 3′-UTR containing miR-2337 MRE (Fig. 2C). This construct was co-treated in HEK293T cells with miR-2337 mimics, and it was found that the overexpression of miR-2337 inhibits the luciferase activity of this construct (Fig. 2D), suggesting that miR-2337 targets the porcine *TGF-β1* gene. Then generated a reporter construct of *TGF-β1* 3′-UTR with haplotype G-G (Fig. 2C), and co-treated HEK293T cells with miR-2337 mimics. Luciferase assay revealed that miR-2337 did not affect the activity of the construct with haplotype G-G (Fig. 2E). Taken together, these results suggest that variants in the MRE motif influence target inhibition of miR-2337 on the *TGF-β1* 3′-UTR in pigs.

### miR-2337 induces, not reduces, TGF-β1 production in GCs

**Fig. 3:**
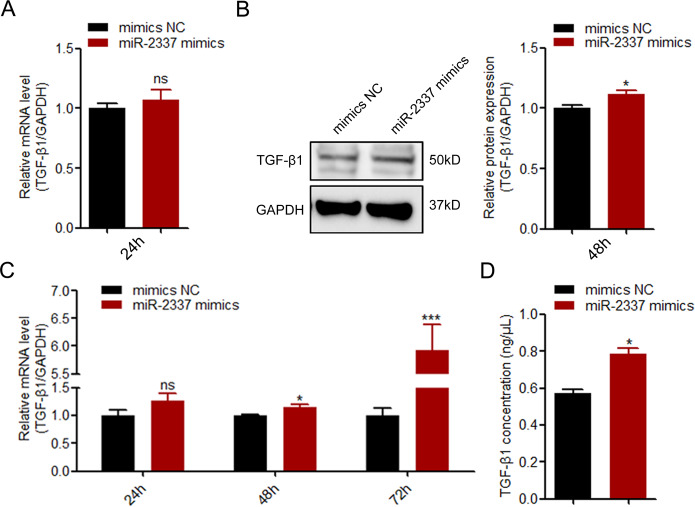
miR-2337 induces TGF-β1 expression in GCs. **A**, **B** GCs were treated with miR-2337 mimics, and mRNA (**A**) and protein (**B**) levels of TGF-β1 were measured at 24 h and 48 h, respectively. **C** GCs were treated with miR-2337 mimics, and TGF-β1 mRNA levels were determined at 24 h, 48 h, and 72 h. **D** TGF-β1 concentration in culture medium of GCs treated with miR-2337 mimics for 48 h, analyzed using ELISA. Data are represented as means ± S.E.M. (*n* = 3). *p < 0.05. ***p < 0.01.

To investigate the regulatory effect of miR-2337 on endogenous *TGF-β1* expression, we analyzed TGF-β1 levels in miR-2337 mimics-treating GCs. After treating with miR-2337 mimics for 24 h, miR-2337 level was significantly increased in porcine GCs (Fig. S4), however, *TGF-β1* mRNA levels were not altered (Fig. 3A). Notably, after miR-2337 mimics treatment for 48 h, TGF-β1 protein level was significantly increased, not decreased (Fig. 3B), indicating that in porcine GCs, miR-2337 is not an inhibitor of TGF-β1. In fact, it may be an activator, as miR-2337 may be a potential saRNA of TGF-β1 in GCs. It is well known that an important feature of saRNA activated gene is the delay in the onset of gene activation [26]. We therefore investigated the kinetics of *TGF-β1* mRNA levels in GC treatment of miR-2337 mimics. As expected, induction of *TGF-β1* transcription by miR-2337 initiate at 48 h (Fig. 3C), indicating that miR-2337 is a potential saRNA of *TGF-β1* in GCs. In addition, we also detected TGF-β1 concentration in culture medium by ELISA, and found that miR-2337 induces upregulation of TGF-β1 production in GCs (Fig. 3D). These data suggest that miR-2337 functions as a potential saRNA of *TGF-β1* to enhance TGF-β1 secretion in GCs.

### miR-2337 is a saRNA of the porcine *TGF-β1* gene in GCs

**Fig. 4:**
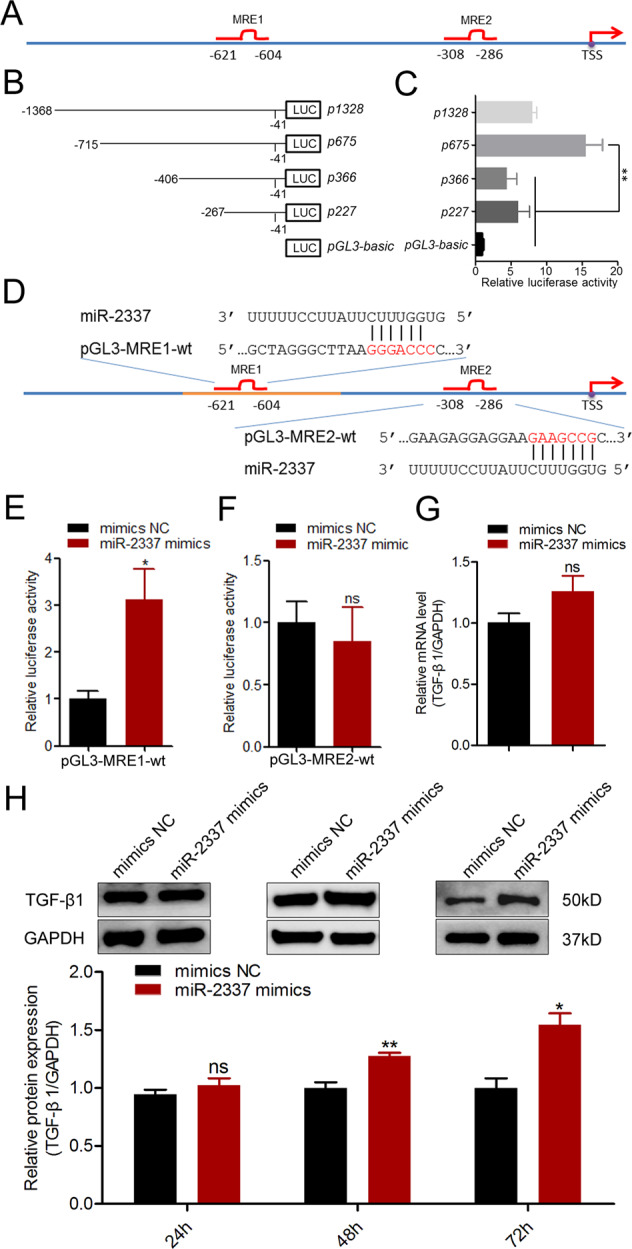
miR-2337 enhances the activity of the *TGF-β1* core promoter. **A** Schematic showing 2000-bp of the promoter region of porcine TGF-β1. Transcription start site (TSS) was considered to be +1. MRE1, miRNA response element 1 for miR-2337. MRE2, miRNA response element 2 for miR-2337. **B**, **C** Identification of the core promoter region. Four deleted constructs p227, p366, p675, and p1328 were generated (**B**), and transfected into GCs. Luciferase assays were measured (**C**). **D** Schematic annotation of the report vector of the TGF-β1 promoter containing the MRE motif. **E**, **F** The effect of miR-2337 on the promoter activity of the TGF-β1 gene. Report vectors of the TGF-β1 promoter containing MRE1 (**E**) or MRE2 (**F**) motif were co-treated with miR-2337 mimics, and luciferase activity was measured. **G**, **H** The effect of miR-2337 on TGF-β1 expression in GCs with the genotype AA/AA/AA. GCs were isolated from sows with the genotype AA/AA/AA and transfected with miR-2337 mimics, TGF-β1 mRNA (**G**) and protein (**H**) level was detected. Data are represented as means ± S.E.M. (*n* = 3). *p < 0.05.

miRNAs have been shown to act as saRNAs, which induce the transcription of target mRNAs by direct interaction with the promoter region of target genes [27]. Therefore, we detected the subcellular localization of miR-2337 in porcine ovarian GCs and showed that the majority of miR-2337 was located in the nucleus and only 10% of which stayed in cytoplasm (Fig. S5). Then we predicted whether the porcine *TGF-β1* promoter region (2000-bp DNA sequences before the transcription start site) contains the MRE motif of miR-2237 by RNAhybrid. Interestingly, two MRE motifs of miR-2237 were detected at −621 to −604 nt (MRE1), and −308 to −286 nt (MRE2) in this region (Figs. 4A and S6). Furthermore, we showed that the MRE1 motif, not the MRE2 motif exists in the core promoter region (−715/−406 nt) (Fig. 4B, C). To further investigate the effect of miR-2337 on core promoter activity, we generated reporter vectors containing the MRE1 motif (Fig. 4D). A luciferase assay revealed that miR-2337 induces an upregulation of the activity of the promoter containing the MRE1 motif (Fig. 4E), indicating that miR-2337 activates the transcriptional activity of the *TGF-β1* gene. However, miR-2337 had no effect on the activity of the promoter containing the MRE2 motif (Fig. 4F), indicating that miR-2337 induces TGF-β1 transcriptional activity via MRE1 motif. Besides, to confirm that MRE motif in the promoter, not in the 3′-UTR is the functional MRE for miR-2337 regulation of TGF-β1 in porcine GCs, we isolated GCs from sows with the AA/AA/AA genotype, which contain a miR-2337 MRE of the TGF-β1 3′-UTR. As expected, there was no significant change in the mRNA level of *TGF-β1* gene in GCs with the AA/AA/AA genotype after 24 h of miR-2337 treatment (Fig. 4G). Furthermore, western blot showed that miR-2337 significantly induced TGF-β1 protein expression in GCs with the AA/AA/AA genotype at 48 h and 72 h, not 24 h after treatment with miR-2337 (Fig. 4H). Taken together, given the *TGF-β1* transcription induced by miR-2337 in GCs, we conclude that miR-2337 is a saRNA of the *TGF-β1* gene in porcine GCs, which induces *TGF-β1* expression via MRE motif in the promoter, not in the 3′-UTR.

### miR-2337 binds to the MRE1 motif to alter histone modification of the *TGF-β1* core promoter

**Fig. 5:**
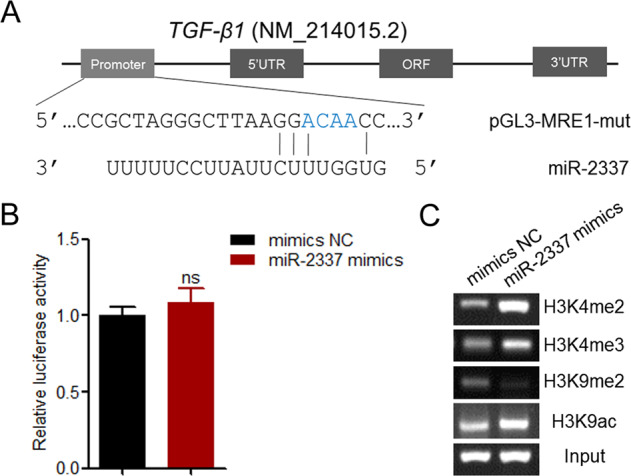
miR-2337 alters histone modification of the *TGF-β1* promoter through direct binds to its MRE1 motif. **A** Schematic annotation of the report vector of the *TGF-β1* core promoter containing the mutant-type MRE1 motif. **B** The effect of miR-2337 on MRE1-mutated core promoter activity. **C** ChIP assay. Changes of histone modifications in the *TGF-β1* core promoter. Data are represented as means ± S.E.M. (*n* = 3). *p < 0.05.

To further investigate whether miR-2337 induces *TGF-β1* transcription via the MRE1 motif, we also constructed a reporter vector of the *TGF-β1* core promoter containing the mutant-type MRE1 motif (Fig. 5A). As expected, miR-2337 did not affect MRE1-mutated core promoter activity (Fig. 5B), indicating that miR-2337 activates *TGF-β1* transcription via the MRE1 motif within its core promoter. It has been shown that saRNA induces target transcription by epigenetic changes in the core promoter region [26]. Therefore, we next tested whether miR-2337 influences histone modification of the *TGF-β1* core promoter in GCs by ChIP assays. Overexpression of miR-2337 induces upregulation of H3K4me2, H3K4me3, and H3K9ac, whereas downregulation of H3K9me2 was induced at the MRE1 motif of the core promoter region (Fig. 5C). Our data suggest that miR-2337 directly binds to the MRE1 motif to alter histone modification of the *TGF-β1* core promoter, thereby activating *TGF-β1* transcription in GCs.

### miR-2337 controls the TGF-β1-mediated TGF-β signaling pathway and GC apoptosis

**Fig. 6:**
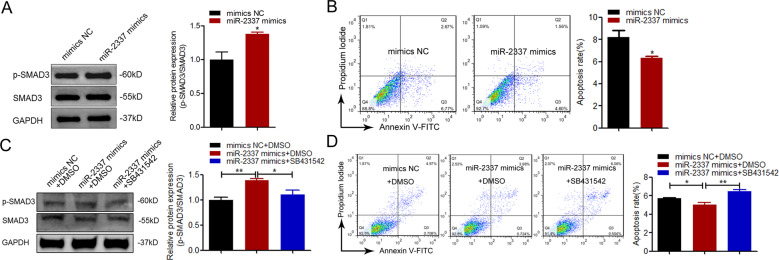
miR-2337 controls the TGF-β1-mediated TGF-β signaling pathway and GC apoptosis. **A**, **B** miR-2337 activates the TGF-β signaling pathway, and inhibits GC apoptosis. Total SMAD3 (t-SMAD3) levels, phosphorylated SMAD3 (p-SMAD3) levels and the apoptosis rate were assessed in miR-2337-overexpressing GCs, using qRT-PCR and flow cytometry. **C**, **D** miR-2337 controls the TGF-β signaling pathway and GCs apoptosis by activating TGF-β1. T-SMAD3 levels, p-SMAD3 levels, and the apoptosis rate were determined in GCs co-treated with SB431542 (an inhibitor of the TGF-β signaling pathway), and miR-2337 mimics, using qRT-PCR and flow cytometry. Data are represented as means ± S.E.M. (*n* = 3). *p < 0.05.

TGF-β1 is both an activator of the TGF-β signaling pathway, and a repressor of porcine GC apoptosis [2]. We therefore tested whether miR-2337 controls the TGF-β signaling pathway and GC apoptosis. Levels of phosphorylated SMAD3 (p-SMAD3), a marker for the activity of this pathway, were significantly increased in miR-2337-overexpressing GCs (Fig. 6A). Flow cytometry showed that miR-2337 overexpression significantly inhibited the GC apoptosis rate (Fig. 6B). These data suggest that, similar to TGF-β1, miR-2337 is both an activator of the TGF-β signaling pathway, and a repressor of GC apoptosis. Furthermore, inactivation of the TGF-β signaling pathway by the TGF-β receptor-specific inhibitor SB431542 inhibited miR-2337 mimics-induced upregulation of p-SMAD3 levels in GCs (Fig. 6C). Consistent with this, miR-2337 could rescue the inactivation of TGF-β signaling pathway caused by TGF-β1 silencing in GCs (Fig. S7). In addition, the inhibitor SB431542 also reversed the downregulation of the GC apoptosis rate caused by miR-2337 mimics (Fig. 6D). These data suggest that miR-2337 controls the TGF-β signaling pathway and cell apoptosis by activating *TGF-β1* in porcine GCs.

## Discussion

TGF-β1 is highly expressed in the reproductive system, which has been shown to be strongly related to female fertility by controlling important events in reproductive cycle, such as, ovulation, fertilization, implantation, early embryo development, and pregnancy [7, 28–30]. In sows, *TGF-β1* is also an important candidate gene for reproductive traits [2, 24]. In this study, we identified three completely linked mutations (c.1583A > G and c.1587A > G, and c.2074A > C) in the porcine *TGF-β1* 3′-UTR, and the TNB of sows with the favorable GG/GG/CC genotype is 0.46 higher than that of other genotypes in the Yorkshire population (*n* = 325). Similarly, in another Yorkshire population (*n* = 567), the TNB of sows carried with the GG/GG genotype for mutations A > G at 2490 nt (i.e., c.1583A > G) and A > G at 2494 nt (i.e., c.1587A > G) was significantly higher than that of the AA/AA genotype [24]. Taken together, these findings suggest that variants in the *TGF-β1* 3′-UTR have a certain impact on the reproductive performance of Yorkshire sows. In the breeding of Yorkshire reproductive traits, these variants in the *TGF-β1* 3′-UTR can be used as potential molecular markers for marker-assisted selection (MAS), that is, to select individuals the favorable genotype GG/GG/CC, so as to quickly improve the fecundity of Yorkshire population.

Mutations in or near RNA regulatory elements have been shown to control the activity or function of the 3′-UTR by affecting the interaction between RNA binding proteins (RBPs), miRNAs and 3′-UTR [31–33]. rs15705, for instance, is identified in the *BMP2* 3′-UTR as an osteoporosis-associated variant in Icelandic patients [34]. This variant disrupts a putative AU-rich element (ARE), which has been shown to influence 3′-UTR activity and posttranscriptional regulation of *BMP2*, perhaps through an ARE motif and RBP interaction mechanism [35, 36]. Interestingly, as a causal variant for natural clearance of infection with HCV infection, variant rs4803217 is located near the three classical ARE motifs and on the MRE motifs of two myomiRs in the IFNL3 3′-UTR, and strongly influences both ARE motif-mediated 3′-UTR activity, and miRNA regulation of 3′-UTR activity [31]. Herein we demonstrated that mutations c.1583A > G and c.1587A > G cause the loss of a miR-2337 MRE motif, thereby relieving the inhibition of miR-2337 on *TGF-β1* 3′-UTR activity. Recently, several mutations in MRE motifs that alter miRNAs interaction with the target 3′-UTR have been identified, such as rs78378222 in the miR-325-3p MRE motif of the *TP53* 3′-UTR [33], rs3802266 in a miR-181a-2-3p MRE motif of the *ZHX2* 3′-UTR [37], and rs41283642 in a miR-142-3p MRE motif of the *TGFBR1* 3′-UTR [38].

Notably, we showed that, in porcine GCs, miR-2337 did, not decrease TGF-β1 levels. Mechanistically, miR-2337 acts as an endogenous saRNA to induce *TGF-β1* transcription and production. saRNAs refer to a double-stranded RNA (dsRNA) with a length of approximately 19–21 nt, which induces target transcription via an RNA activation (RNAa) mechanism by directly interacting with the promoter region of target genes [27, 39, 40]. Since 2006, scientists have found that 21 nt of dsRNA that are complementary to the promoter of targets, such as p21 and VEGF, can continuously induce target transcription in human cells. SaRNA has become a novel therapeutic, and has been used in clinical trials [41, 42]. Dozens of synthetic saRNAs have been identified to activate target transcription, for example, saRNAs KLF4-PR1, MYC-PR1, and MYC-PR2 activate KLF4 and c-MYC, two pluripotency reprogramming factors in mesenchymal stem cells [43]. Moreover PR11 [39] and PR-1611 [40] activate the *PR* gene in breast cancer cells, and CEBPA-saRNA activates the *CEBPA* gene in hepatocellular carcinoma cells [44]. Interestingly, in 2008, miRNAs, a class of naturally occurring endogenous small ncRNAs, have also been shown to function as endogenous saRNAs to directly activate target expression [45]. At present, several miRNAs that mediate the activation of targets have been identified, including miR-24-1 [46], miR-4281 [47], and miR-320 [48]. Taken together, our findings identified the first saRNA target *TGF-β1* and TGF-β superfamily, which provides new insight into miRNs regulation of TGF-β1. However, the mechanism of the variants in the *TGF-β1* 3′-UTR in terms of affecting sow reproductive performance requires further study.

Crosstalk between miRNAs and the TGF-β signaling pathway is one of the most important regulatory mechanisms of GC apoptosis in mammals [49, 50]. On the one hand, the TGF-β signaling pathway controls GC functions by regulating miRNA biosynthesis. For example, miR-224/320/383-mediated mouse GC proliferation and estradiol release [49, 51], miR-10a/10b-mediated GC states in humans and rodents [52], and miR-1306/143/29c/425-mediated porcine GC apoptosis [13, 50, 53, 54] are all regulated by the TGF-β signaling pathway. On the other hand, miRNAs directly target core members of other ligands, receptors, or SMADs of the TGF-β signaling pathway to regulate GC functions. For ligands, miR-130a induces GC apoptosis by reducing *TGF-β1* in pigs [2]. For receptors, let-7g and miR-202-5p induce GC apoptosis by inhibiting pig *TGF-β1* and goat *TGF-β1*, respectively [55, 56]. For SMADs, miR-4110 promotes goat GC apoptosis by inhibiting *SMAD2* [57], and miR-224 controls mouse GC proliferation and estradiol release by inhibiting *Smad4* [50]. Herein, we showed for the first time that miRNA activates the TGF-β signaling pathway to reduce GC apoptosis by target directly activating a core component of this pathway. Our findings defined the new function of miR-2337, and identify this miRNA as an activator of the TGF-β signaling pathway in GCs.

**Fig. 7:**
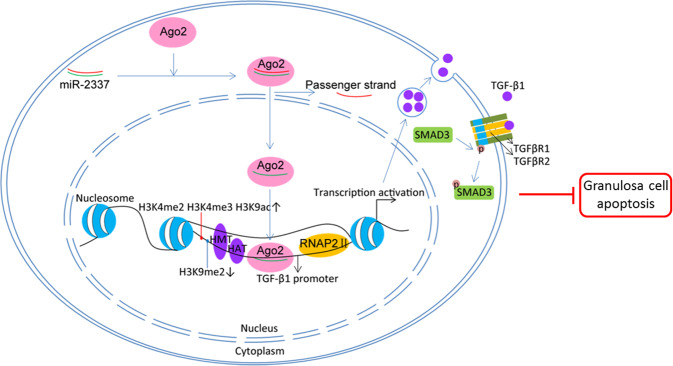
Schematic showing the mechanisms underlying miR-2337 induction of TGF-β1 production in GCs. miR-2337 direct binds to the MRE motif of TGF-β1 promoter and then alters its histone modification, to activate TGF-β1 transcription and induce TGF-β1 production, thereby inhibit GC apoptosis through activating the TGF-β signaling pathway.

In conclusion, we screened the whole 3′-UTR of the porcine *TGF-β1* gene and show the important role that *TGF-β1* 3′-UTR variants play on Yorkshire sow fertility. Importantly, mutations c.1583A > G and c.1587A > G that are located at the MRE motif of miR-2337 in the *TGF-β1* 3′-UTR, relieves the inhibition of miR-2337 on 3′-UTR activity. However, in porcine GCs, miR-2337 does not reduce transcription and production of TGF-β1. We identified miR-2337 as a saRNA of *TGF-β1* that induces *TGF-β1* transcription through direct binding to the promoter and alterations in histone modification (Fig. 7). Furthermore, miR-2337 activates the TGF-β signaling pathway and reduces GC apoptosis by activating *TGF-β1*. Our findings implicate that miR-2337 might be an activator of the TGF-β signaling pathway, and a promising therapeutic target for GC apoptosis and low fertility.

## Methods

### Samples and ethics

A total of 365 sows were included in the present study. The studied population was randomly selected from Jiangsu Kangle Pig Breeding Farm (Yorkshire sows, *n* = 325), Jiangsu Huaiyin Pig Breeding Farm (Suhuai sows, *n* = 20) and Changzhou Erhualian Production Cooperation (Erhualian sows, *n* = 20) for ear tissue sample collection and genomic DNA extraction. Their corresponding reproductive trait records were also collected for further association analysis. In addition, ovaries were isolated from 60 Duroc-Landrace-Yorkshire sows (sexually mature) for GC collection and culturing. In this study, all animal experiments were reviewed and supervised by the Animal Ethics Committee (AEC) of Nanjing Agricultural University, Jiangsu, China.

### Genomic DNA isolation and genotyping

Using the phenol-chloroform method as previously described [2], the genomic DNA from ear samples of the studied Yorkshire sows was extracted and stored at −20 °C for further analyses. After detection of the concentration, purity and integrity of the DNA, a total of 325 DNA samples were selected for genotype validation. Using genomic DNA as a template, the variants in the 3′-UTR region of porcine *TGF-β1* were amplified by PCR and identified using Sanger sequencing procedures (Sangon Biotech, Shanghai, China). The specific primers used for genotype validation are listed in Table S1.

### Cell culture and treatment

Porcine GCs were extracted from small non-atretic follicles using a 22-gague sterile syringe, and washed twice with 37 °C PBS. Next, the cells were seeded in culture plates filled with Dulbecco’s modified Eagle’s medium (DMEM/F12, #2177608, Gibco) containing 15% fetal bovine serum (FBS, #10270106, Gibco) and 1% penicillin/streptomycin solution (PS, #15140-122, Gibco), which was subsequently placed in a humid incubator at 37 °C and 5% CO_2_ aerobic conditions. KGN and HEK293T cells were seeded in plates filled with RPMI-1640 medium (#AF29477380, Hyclone) and DMEM medium (#AF29431640, Hyclone) containing 10% FBS and 1% PS, and cultured under the same conditions as porcine GCs. For transfection of recombinant plasmids and oligonucleotides, HighGene transfection reagent (#96191114TR, ABclonal) was used following the manufacturer’s instructions. The oligonucleotides, siRNA [58] and mimics used in this study are presented in Table S2. For the TGF-β inhibitor treatment, first, using non-FBS medium cultured for 10 h in order to starve GCs. Next, SB431542 was diluted and added to the medium to achieve a final concentration of 50 μM.

### Bioinformatics analysis and association analysis

The full length of the porcine *TGF-β1* nucleotide sequence was downloaded from the Ensembl database (http://asia.ensembl.org/index.html). To predict the putative promoter of porcine *TGF-β1*, two online software, namely Promoter scan and Promoter 2.0, with different algorithms were utilized according to their corresponding instructions. In addition, the candidate miRNAs that potentially target porcine *TGF-β1* were predicted using the miRBase database (https://www.mirbase. org/search.shtml), and the MREs of miR-2337 within the promoter and the 3′-UTR region of porcine *TGF-β1* were analyzed using RNAhybrid (https://bibiserv.cebitec.uni-bielefeld.de/rnahybrid). The genome location and sequence of mature miR-2237 were obtained from the NCBI database (https://www.ncbi.nlm.nih.gov/gene/?term=miR-2337). Furthermore, association analyses were performed in this study to detect the correlation between polymorphism of the variants within the 3′-UTR of porcine *TGF-β1* and reproductive traits, such as the total number of piglets born (TNB) and the number of piglets born alive (NBA). SAS software was utilized with the general linear model as described in our previous study [2].

### Plasmids and dual-luciferase reporter assay

To identify the core promoter of porcine *TGF-β1*, different fragments of the *TGF-β1* promoter were amplified and inserted into pGL3-basic vectors (#E1751, Promega) between *Xho*I and *Hind*III restriction enzyme sites. To detect whether miR-2337 directly targets *TGF-β1*, the 3′-UTR fragment of porcine *TGF-β1*, which contains a putative MRE of miR-2337 was synthesized and cloned into the pmirGLO vector (#E1330, Promega) between *Nhe*I and *Xba*I restricted enzyme sites. To analyze the effects of miR-2337 on the promoter activity of *TGF-β1*, the promoter of porcine *TGF-β1* containing a potential MRE of miR-2337 was cloned into a pGL3-basic vector between the *Sac*I and *Hind*III restricted enzyme sites. Trelief^TM^ SoSoo Cloning kit (#TSV-S1, TsingKe) was used to generate mutant-type reporter vectors following the manufacturer’s instructions. All recombinant vectors were verified by Sanger sequencing (Sangon Biotech, Shanghai, China). The primers used for plasmid construction are shown in the Table S3. For dual-luciferase reporter assay, cells were lysed and the content was collected after treatment for 24 h. Next, the luciferase activities of *Firefly* and *Renilla* were detected using a Luciferase Activity Detection System kit (#32981788, Promega), and the ratio of *Firefly*/*Renilla* was used to determine the relative luciferase activity of each sample.

### RNA extraction and quantitative real-time PCR

Total RNA from porcine GCs under different treatments was extracted using Trizol reagent (#RG-51001A, Angle Gene, China), and cDNA was synthesized using PrimeScript^TM^ RT Master Mix (#RR036A, TaKaRa, China) with 1 μg of total RNA as the template according to the manufacturer’s instructions. qRT-PCR was performed using the Power SYBR Green PCR Master Mix (#Q111-02/03, Vazyme) and the reactions were detected on a StepOne Plus System (Applied Biosystems) as previously published [13]. The fold changes of the genes of interest were calculated using the 2^-ΔΔCT^ method, and the relative expression levels of coding and non-coding genes were normalized to the levels of *GAPDH* and *U6*, respectively. The primers designed for the qPCR analysis are listed in Table S4.

### Western blotting

Porcine GCs were lysed using RIPA lysis buffer (#BD0031, Bioworld) with 1% PMSF and protease inhibitor, and the total protein was extracted and collected for western blotting. The concentration of each protein sample was detected using the BCA method according to the manufacturer’s instructions. In brief, equal amounts of protein (~20 μg) from different samples were uploaded and separated by 4–20% SDS-PAGE. After electrophoresis for 1 h at 140 V, the separated proteins were transferred to PVDF membranes. The PVDF membranes were then incubated with blocking buffer containing 5% BSA and probed with the primary antibodies overnight. The next day, the membranes were resined with TBST buffer three times to remove excess primary antibodies, and then incubated with HRP-conjugated secondary antibodies. The high-solution images were obtained using a chemiluminescence imaging system, and the grayscale of each protein blot was quantified using ImageJ software. GADPH protein expression served as an internal control. The primary antibodies used were TGF-β1 (#bs-0086R, Bioss, 1:1000 dilution), SMAD3 (#D155234, Sangon, 1:1000 dilution), p-SMAD3 (#D155153, Sangon, 1:1000 dilution), and GAPDH (#D198662, Sangon, 1:2000 dilution).

### ELISA

In this study, ELISA was performed to detect the secretion levels of TGF-β1 in the culture medium of GCs. In brief, after 48 h of treatment with miR-2337, the cell culture medium was collected. The activator was used in the ELISA kit to activate TGF-β1, 100 µL of standard and samples were added to the wells, and the mixture was allowed to incubate for 90 min with dry spinning. Next, in the following sequence, biotinylated antibody, washing solution, enzyme conjugate working solution, substrate solution, and working solution were added and processed. Finally, the optical density (OD) value of each well was measured using a microplate reader at 450 nm wavelength.

### Subcellular localization

Nuclear and cytoplasmic isolation was performed using the method described by Du et al. [59]. Briefly, 1 mL of lysate buffer was added to a centrifuge tube containing porcine GCs, lysed for 5 min, and then centrifuged for 3 min (10,000 × g), and the supernatant containing cytoplasmic extract was absorbed and stored at −80 °C. The nuclear pellet was resuspend and then centrifuge for 3 min (10,000 × g), discard the supernatant, and the nuclear extract is present in the pellet. RNA extraction and qRT-PCR was carried out by the above methods.

### ChIP assay

ChIP assays were performed using Pierce Agarose ChIP Kit (#26156, Thermo) according to the manufacturer’s protocol. In brief, after transfection for 72 h, 1% formaldehyde was added to the medium and porcine GCs were cross-linked for 10 min, and then under 6U of micrococcal nuclease had been added in a 37 °C water bath for 15 min. H3K4me2 antibody (#9725 S), H3K4me3 antibody (#9751 S), H3K9me2 antibody (#4658 S) and H3K9ac antibody (#9649 S) were all obtained from Cell Signaling Technology (USA), and were used to pull down the immunoprecipitated complex. After pull down, the fragments of interest were detected by PCR and the amplified products were analyzed by 3% agarose gel electrophoresis, and the enrichment was normalized to the IgG group. IgG antibody (#2985 S, Cell Signaling Technology) was used as a negative control and unprocessed DNA served as the input control. The primers used for the ChIP assay are listed in Table S5.

### Apoptosis detection

After 48 h of treatment, porcine GCs were collected and the cell apoptosis rate was detected using a Cell Apoptosis Detection kit (#A211-01/02, Vazyme). First, porcine GCs were harvested and rinsed with cold PBS twice after treatment for 48 h, then 100 μL of binding buffer were added to resuspend the cells for 10 min. Subsequently, 5 μL Annexin V-FITC and 5 μL propidium iodide (PI) were added to dye cells for at least 10 min under dark conditions. Total GCs were detected using fluorescence-activated cell sorting (FACS) on a cell sorting machine, and the apoptosis rate was analyzed using FlowJo software.

### Statistics analysis

In this study, data were analyzed using GraphPad Prism v5.0 software, and represented as mean ± S.E.M. with at least three independent replicates. The differences (*p-*values) between two different groups were determined by a two-tailed Student’s *t* test, and *p* < 0.05 was considered a statistically significant difference.

## Supplementary information

Supplemental data

## Data Availability

The datasets used and analyzed during the current study are available from the corresponding author on reasonable request.
