# Supplementary material for: miR-2337 induces TGF-β1 production in granulosa cells by acting as an endogenous small activating RNA
Source: Cell Death Discov. 2021 Sep 18;7:253. doi: 10.1038/s41420-021-00644-4 (PMC8449777; doi:10.1038/s41420-021-00644-4)
Supplement: Supplementary file 1 — Supplemental data [file 41420_2021_644_MOESM1_ESM.doc]

**Supplemental data**

**Supplemental Figure legends**

**Fig. S1. Peak plot of pooled-DNA sequencing of Yorkshire, Suhuai and Erhualian sows.**

**Fig. S2. The binding capacity between miR-2337 and the 3’-UTR of the porcine *TGF-β1* gene.**

**Fig. S3. The Sequence of the 3’-UTR of the porcine *TGF-β1* gene.** Transcription start site was assumed to be +1 (GenBank ID: XM_021093503.1). Underlining indicate RNA regulatory elements (RREs), such as miRNA response elements (MREs), AU-rich elements (AREs), GU-rich elements (GREs), and polyA signals (PAS). Arrow represents three mutations: c.1583A>G, c.1587A>G, and c.2074A>C.

**Fig. S4. The expression levels of miR-2337 in porcine GCs treated with miR-2337 mimics.** Data are represented as means ± S.E.M. (n = 3). ** *p* < 0.01.

**Fig. S5. Subcellular localization assay of miR-2337 in porcine ovarian GCs.**

**Fig. S6. The sequence of the promoter region of the porcine *TGF-β1* gene.** Transcription start site was assumed to be +1 (GenBank ID: XM_021093503.1). Underlining indicates miRNA response elements (MREs) of miR-2337. The blue font indicates the sequence of the core promoter region of the *TGF-β1* gene.

**Fig. S7. miR-2337 rescues the inactivation of TGF-β signaling pathway caused by TGF-β1 silencing in GCs.** (A) TGF-β1 mRNA level was detected in GCs treated with TGF-β1-siRNA. (B) The effect of miR-2337 on the expression of p-SMAD3 protein in GCs after *TGF-β1* silencing. Data are represented as means ± S.E.M. (n = 3). * p < 0.05. ** p < 0.01.

**Fig. S1**

**
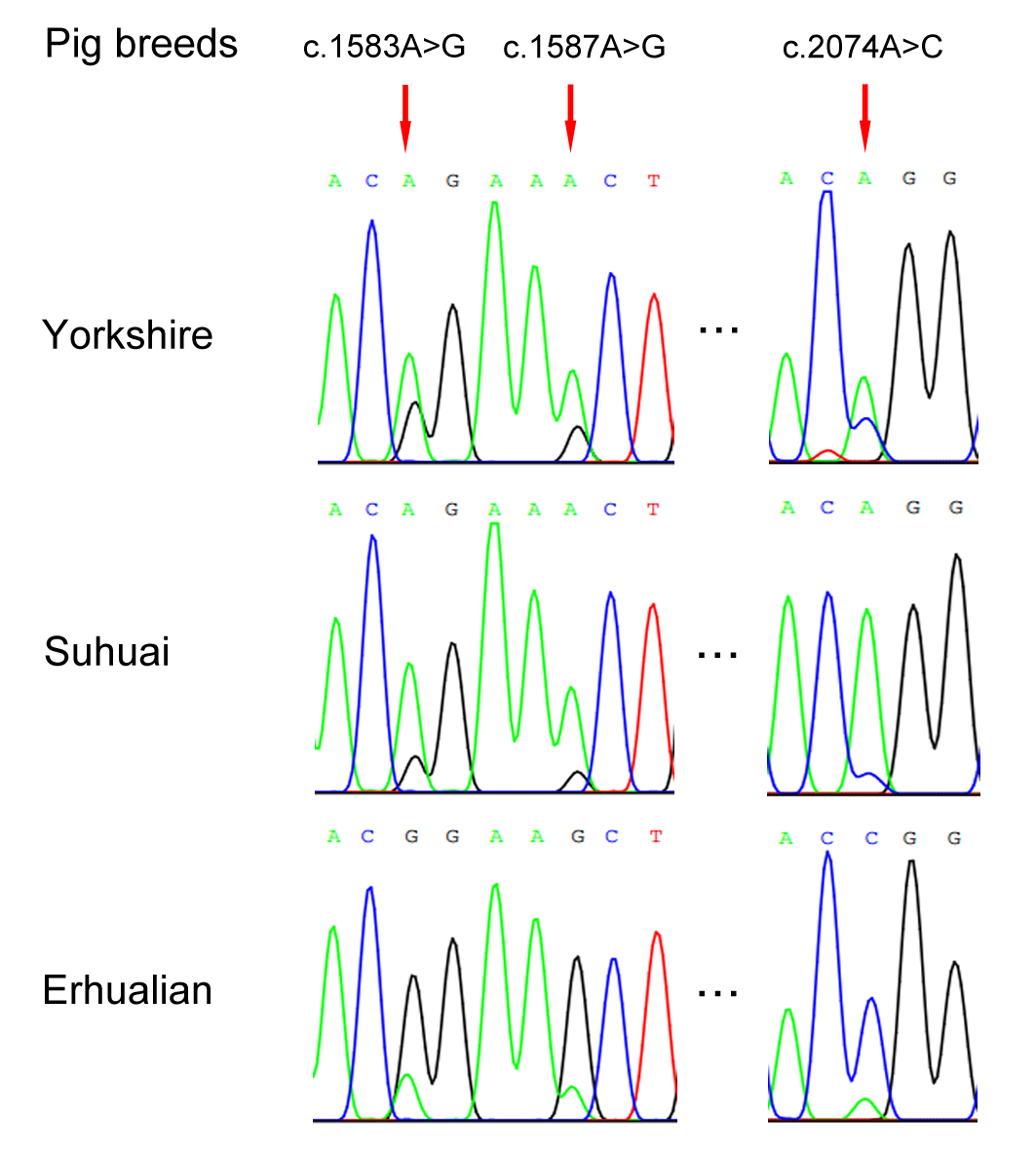
**

**Fig. S2**


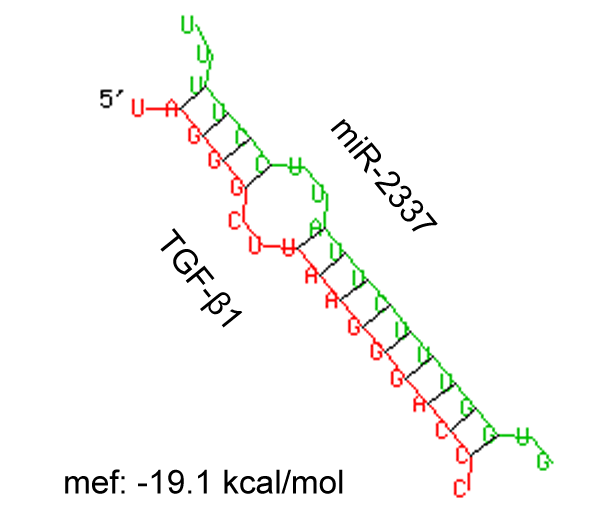


**Fig. S3**

**
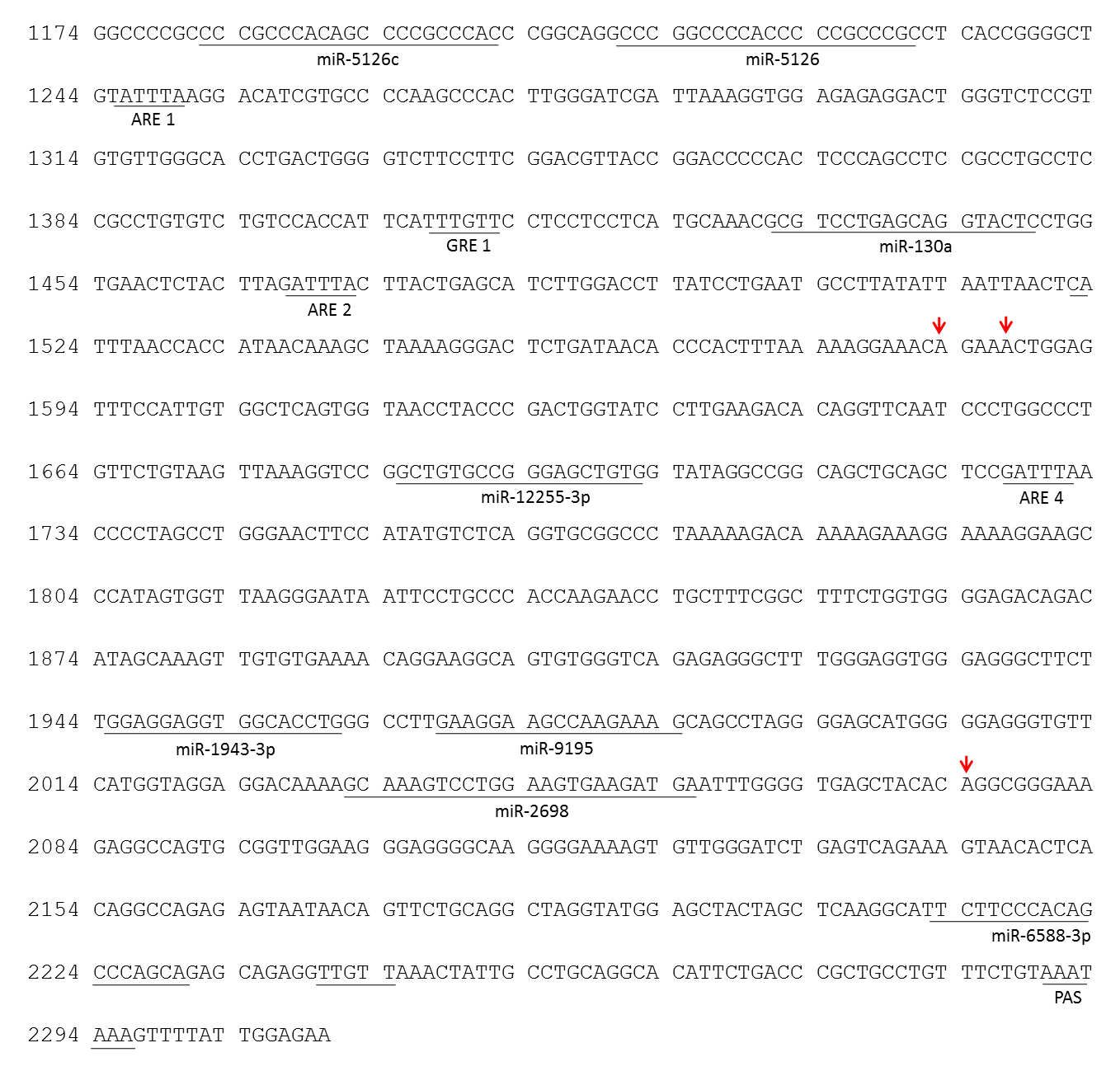
**

**Fig. S4**


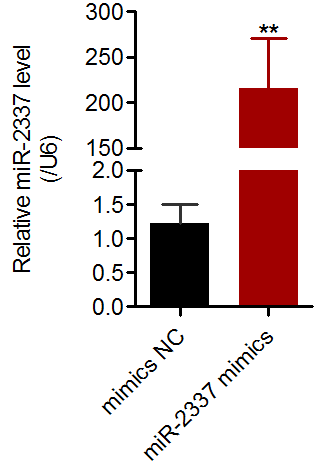


**Fig. S5**


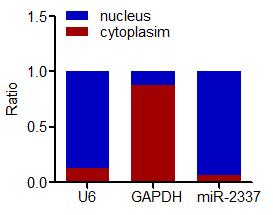


**Fig. S6**


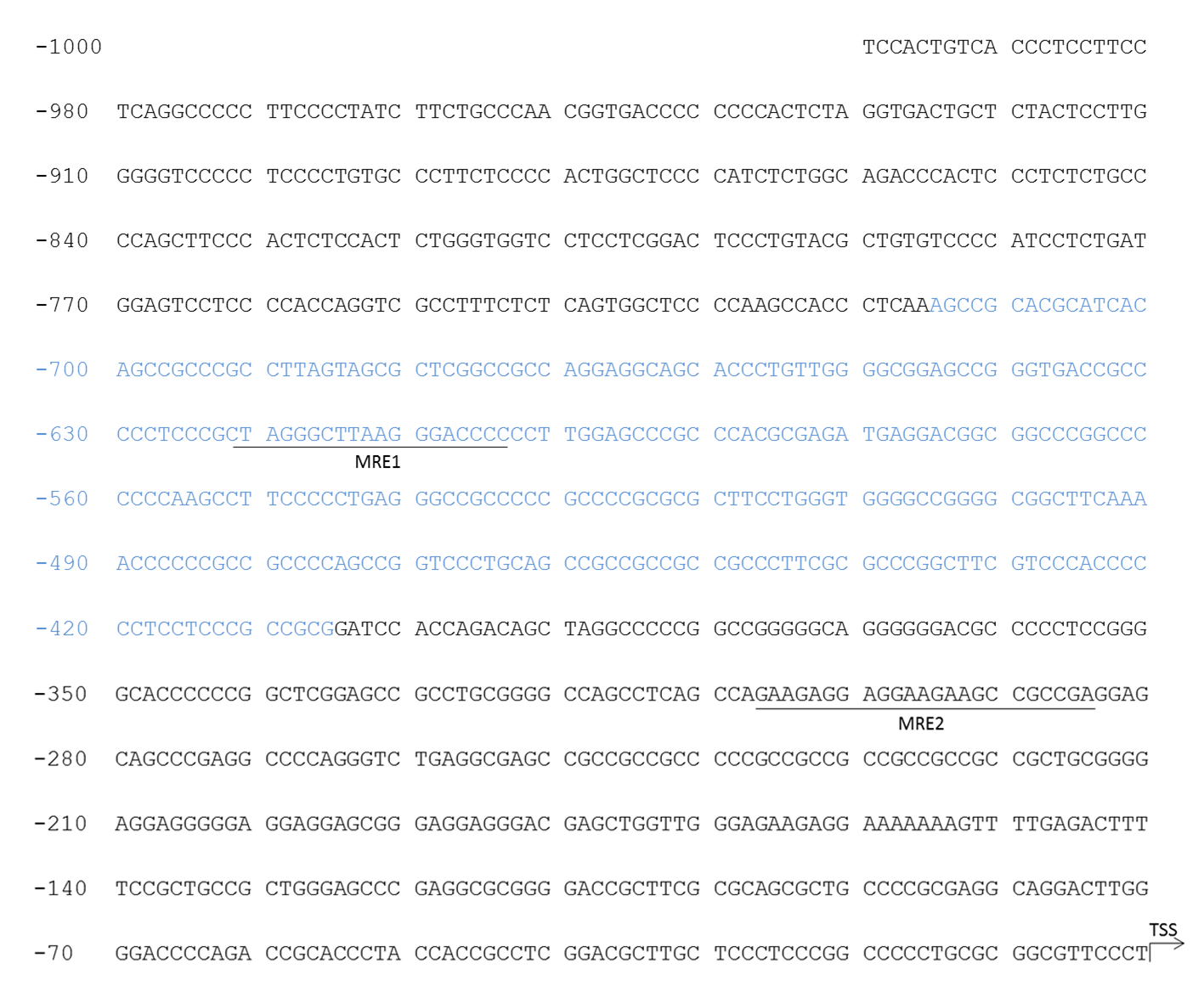


**Fig. S7**


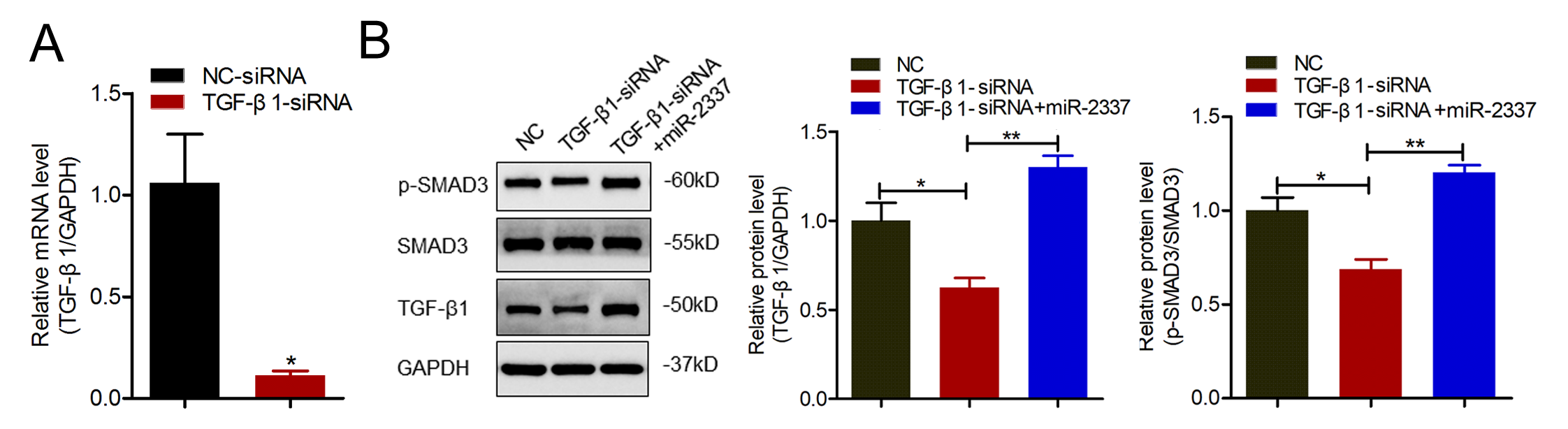


**Supplemental Tables**

**Table S1**

**primers used for genotyping**

| **Name** | **Primer sequence (5’ to 3’)** | **Annealing temp (℃)** | **Product size (bp)** |
| --- | --- | --- | --- |
| TGF-β1 | F: TGTCCACCATTCATTTGTTC | 59 | 714 |
|  | R: CTCCCTTCCAACCGCACT |  |  |

**Table S2**

**RNA oligos** in this study

| Name | Sequences |
| --- | --- |
| mimics NC | UUCUCCGAACGUGUCACGUTT |
|  | ACGUGACACGUUCGGAGAATT |
| miR-2337 mimics | GUGGUUUCUUAUUCCUUUUU |
|  | AAAGGAAUAAGAAACCACUU |
| siRNA NC | UUCUCCGAACGUGUCACGUTT |
|  | ACGUGACACGUUCGGAGAATT |
| TGF-β1-siRNA | CCAGAAAUACAGCAAUGAUTT |
|  | AUCAUUGCUGUAUUUCUGGTT |

**Table S3**

**Primers used for plasmids construction**

| **Name** | **Primer sequence (5’ to 3’)** | **Annealing temp (℃)** | **Product size (bp)** |
| --- | --- | --- | --- |
| pmirGLO-  TGF-β1 | F: CTAGCTAGCAGCCACTGCCCATCGTGTACTA | 68 | 838 |
|  | R: TGCTCTAGACTGCCTTCCTGTTTTCACACAACT |  |  |
| p1327 | F: CCGCTCGAGCGACCGCTACGGGGTGGAAT | 66 | 1327 |
|  | R: CCCAAGCTTGCGGTGGTAGGGTGCGGTCT |  |  |
| p674 | F: CCGCTCGAGAAAGCCGCACGCATCACAGC | 66 | 675 |
|  | R: CCCAAGCTTGCGGTGGTAGGGTGCGGTCT |  |  |
| p383 | F: CCGCTCGAGGCGGATCCACCAGACAGCTAG | 66 | 383 |
|  | R: CCCAAGCTTGCGGTGGTAGGGTGCGGTCT |  |  |
| p244 | F: CCGCTCGAGCCCAGGGTCTGAGGCGAGC | 66 | 244 |
|  | R: CCCAAGCTTGCGGTGGTAGGGTGCGGTCT |  |  |
| pGL3-MRE1*-*wt | F: CGAGCTCAAAGCCGCACGCATCACAGC | 68 | 352 |
|  | R: CCCAAGCTTGGGGGCCTAGCTGTCTGGTG |  |  |
| pGL3-MRE1-mut | F: CTTAAGGACAACCCCTTGGAGCCCGCCCACGCG | 68 | 5128 |
|  | R: CAAGGGGTTGTCCTTAAGCCCTAGCGGGAGGGG |  |  |
| pGL3-MRE2-wt | F: CCGCTCGAGGCGGATCCACCAGACAGCTAG | 66 | 383 |
|  | R: CCCAAGCTTGCGGTGGTAGGGTGCGGTCT |  |  |

**Table S4**

Primers used for qRT-PCR

| **Name** | **Primer sequence (5’ to 3’)** | **Annealing temp (℃)** | **Product size (bp)** |
| --- | --- | --- | --- |
| *TGF-β1* | F: GGCACCCCCCACAGCTTATAT | 60 | 291 |
|  | R: GTGGGCACTGAGGCGAAAAC |  |  |
| *GAPDH* | F: GGACTCATGACCACGGTCCAT | 60 | 220 |
|  | R: TCAGATCCACAACCGACACGT |  |  |
| *miR-2337* | F: CGGGCGTGGTTTCTTATT | 60 | 56 |
|  | R: CAGCCACAAAAGAGCACAAT |  |  |
| *U6* | F: GCTTCGGCAGCACA TATACT | 60 | 56 |
|  | R: TTCACGAATTTGCGTGTCAT |  |  |

**Table S5**

Primers used for ChIP assay

| **Name** | **Primer sequence (5’ to 3’)** | **Annealing temp (℃)** | **Product size (bp)** |
| --- | --- | --- | --- |
| ChIP | F: CCCCACCAGGTCGCCTTTCT | 60 | 193 |
|  | R: CGCCGTCCTCATCTCGCGT |  |  |
